# Assessing the Efficacy of Botulinum Toxin in Treating Neuropathic Pain: A Report of Two Cases

**DOI:** 10.7759/cureus.80249

**Published:** 2025-03-08

**Authors:** Letícia L Cruz, Sara J Mota, Isabel P Catarino, Helder P Cardoso

**Affiliations:** 1 Anesthesiology, Unidade Local de Saúde do Tâmega e Sousa, E.P.E., Penafiel, PRT; 2 Orthopedics, Unidade Local de Saúde do Tâmega e Sousa, E.P.E., Penafiel, PRT

**Keywords:** botulinum toxin a, neuropathic pain, peripheral nerve injury, refractory pain management, surgical neurotomy

## Abstract

This study examines the efficacy of botulinum neurotoxin type A (BoNT-A) in neuropathic pain management. Neuropathic pain (NP) is a chronic condition that remains challenging to treat. While botulinum neurotoxin type A (BoNT-A) has been proposed as a therapeutic option, its efficacy in trauma-induced NP remains unclear. This report presents two patients with NP in their fingers - one following a traumatic amputation and the other a sharp laceration - who underwent two sessions of BoNT-A injections. Neither patient experienced improvement in pain severity or functionality, and severe allodynia persisted. These findings suggest that BoNT-A may have limited efficacy in certain neuropathic pain conditions, emphasizing the need for careful patient selection and further research into alternative therapeutic strategies. Additionally, this study introduces the "neuropathic finger" as a model for investigating local drugs with potential antineuropathic properties.

## Introduction

Neuropathic pain (NP) is a complex and debilitating condition caused by damage or disease affecting the somatosensory nervous system, often presenting as chronic pain with burning sensations, tingling, and electric shock-like pain, significantly impairing quality of life [1]. Despite the availability of several pharmacological treatments, these often provide incomplete pain relief and are associated with adverse effects [2]. Botulinum neurotoxin type A (BoNT-A), derived from *Clostridium botulinum*, has been proposed as a therapeutic option due to its ability to inhibit neurotransmitter release by targeting proteins essential for acetylcholine (ACh) release [3,4]. While BoNT-A is FDA-approved for conditions such as cervical dystonia, blepharospasm, strabismus, hemifacial spasm, hyperhidrosis, and chronic migraine, its efficacy in NP remains unclear [3,5]. Growing evidence suggests potential benefits in conditions such as postherpetic neuralgia, trigeminal neuralgia, complex regional pain syndrome, and diabetic neuropathy, with administration routes varying based on indication, including intradermal, intramuscular, and perineural injections [3,5]. Beyond its effects on ACh release at the neuromuscular junction, BoNT-A is thought to relieve NP through suppression of pain mediators (e.g., substance P, glutamate, and CGRP), reduction of neurogenic inflammation, retrograde axonal transport, and modulation of sodium and calcium ion channels [2]. NP presents unique research challenges due to its diverse mechanisms, broad distribution, and overlap with other pain types, making it difficult to pinpoint pain generators or optimize drug delivery [6]. Given these complexities, this study introduces the "neuropathic finger model" as a potential research platform for studying NP, providing a localized and accessible approach for evaluating investigational antineuropathic drugs. This study examines BoNT-A administration in two patients with trauma-induced NP and aims to contribute to the existing knowledge of its clinical efficacy.

## Case presentation

Case 1

**Figure 1:**
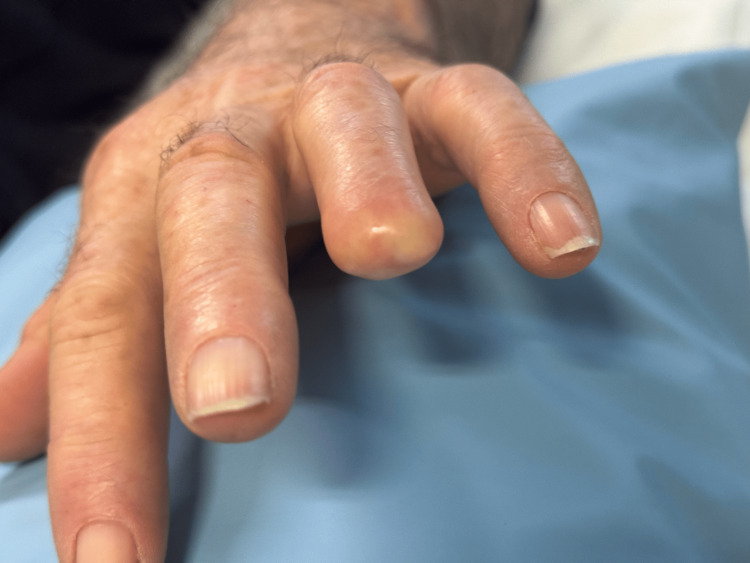
Neuropathic finger after traumatic amputation in Case 1 patient.

A 53-year-old construction worker presented to the chronic pain unit with refractory neuropathic pain. The pain developed following a traumatic amputation of the distal tip of the fourth finger, which had occurred four months earlier. It was characterized by severe allodynia, a stinging sensation, and spontaneous paroxysmal pain described as electric shocks. Pain intensity was rated as 4 at rest and 10 with light touch, significantly impairing hand function and preventing the patient from working (Figure 1).

The patient was treated with paracetamol 1 g every 8 h, pregabalin 200 mg every 12 hours, and tramadol 50 mg every 8 h; however, this regimen was insufficient to alleviate pain or restore functionality for a return to work. Additionally, the treatment was associated with significant side effects, including lightheadedness, constipation, and daily sleepiness, leading the patient to discontinue therapy.

Case 2

A 44-year-old patient presented with chronic neuropathic pain persisting for six months following a linear laceration to the distal phalanx of the index finger from a kitchen knife injury. The pain characteristics mirrored those in Case 1, including severe allodynia, spontaneous electric shock-like pain, and stinging sensations. Pain intensity was rated as 3 at rest and 7 with light touch or hand manipulation, significantly impairing daily activities. The patient was treated with paracetamol 1 g every 8 hours, pregabalin 200 mg twice daily, and amitriptyline 10 mg at night; however, the treatment was ineffective, leading to gradual discontinuation of the medication.

**Figure 2:**
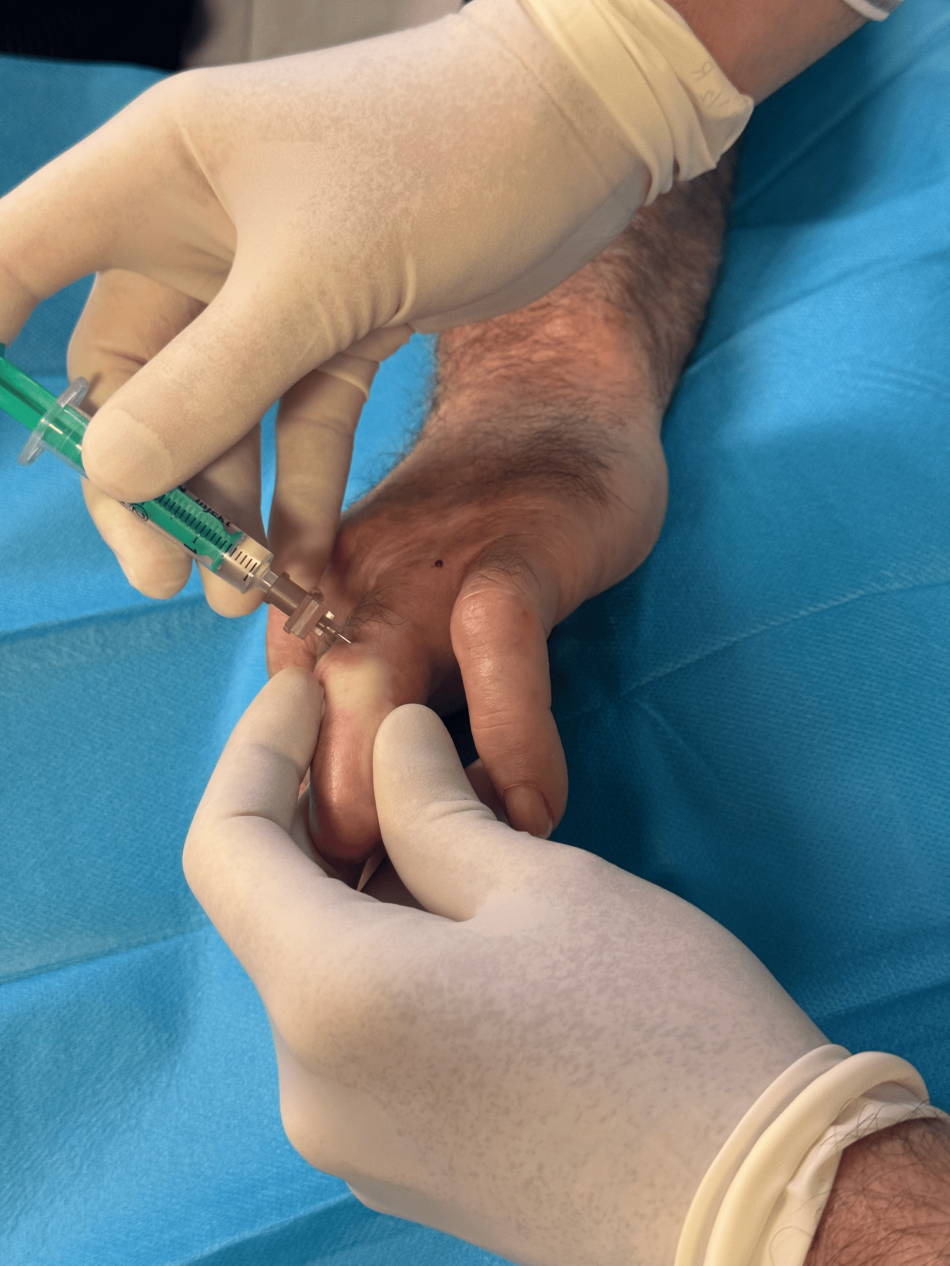
Injection of botulinum toxin type A in Case 1 patient.

Due to pharmacologic treatment failure, both patients were selected for BoNT-A injections. Each received two treatment sessions, spaced eight weeks apart after the first session proved ineffective. In the initial session, 50 units were administered (25 units to the medial and lateral sides of the affected fingers). In the second session, the dose was increased to 100 units (50 units per side). The injection point was at the most proximal part of the painful area, with the injected substance manually squeezed distally to enhance drug distribution toward the fingertip (Figure 2).

**Figure 3:**
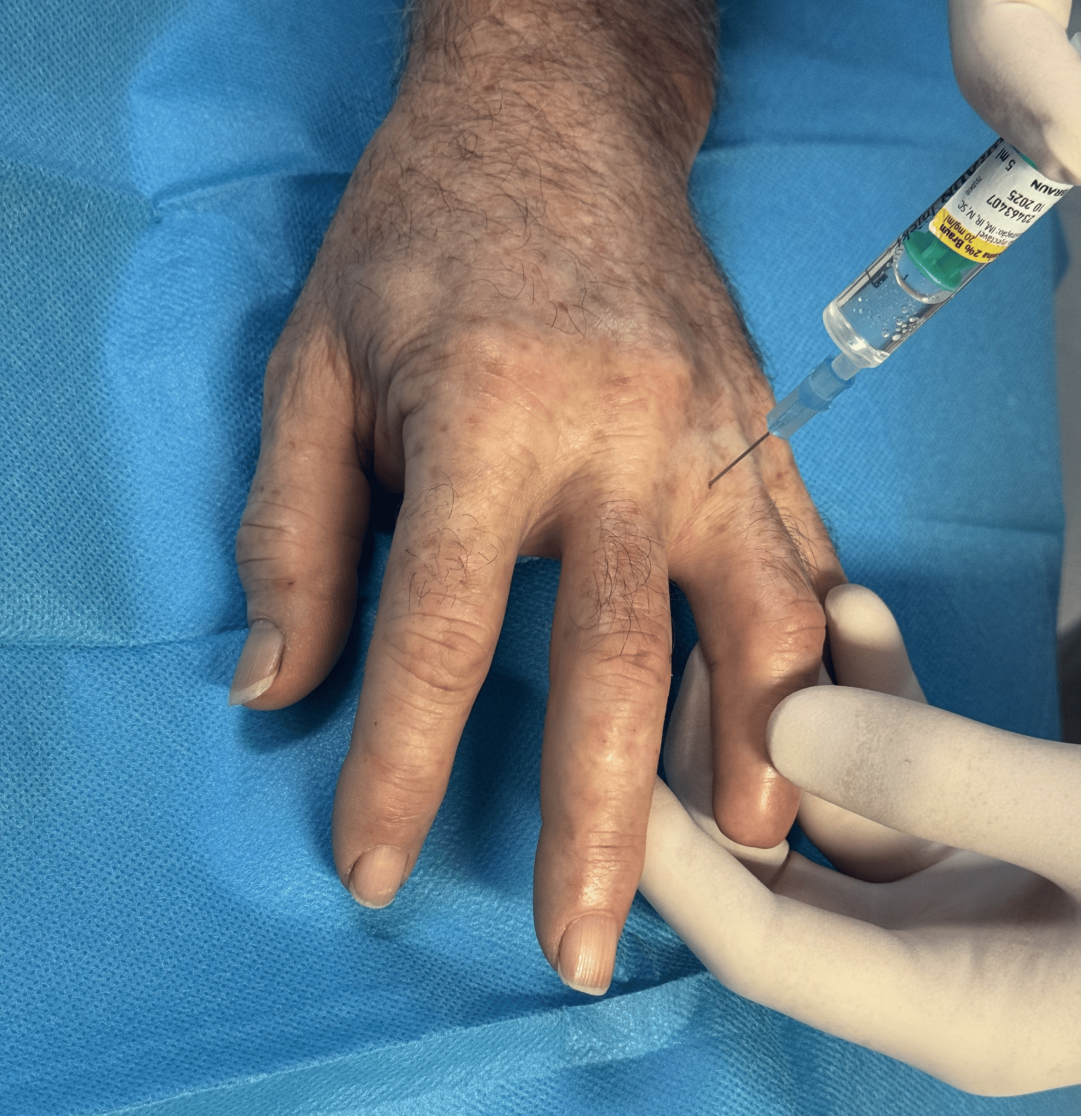
Digital nerve block performed before botulinum toxin injection into the painful area in Case 1 patient.

For patient comfort, a digital nerve block was performed in both patients before injecting botulinum toxin into the painful areas. This was achieved by administering 3 mL of 2% lidocaine on each side of the finger near the metacarpophalangeal joint. The nerve block was necessary due to the low compliance of soft tissues in the fingers, which could otherwise cause intense pain during botulinum toxin injection (Figure 3).

**Figure 4:**
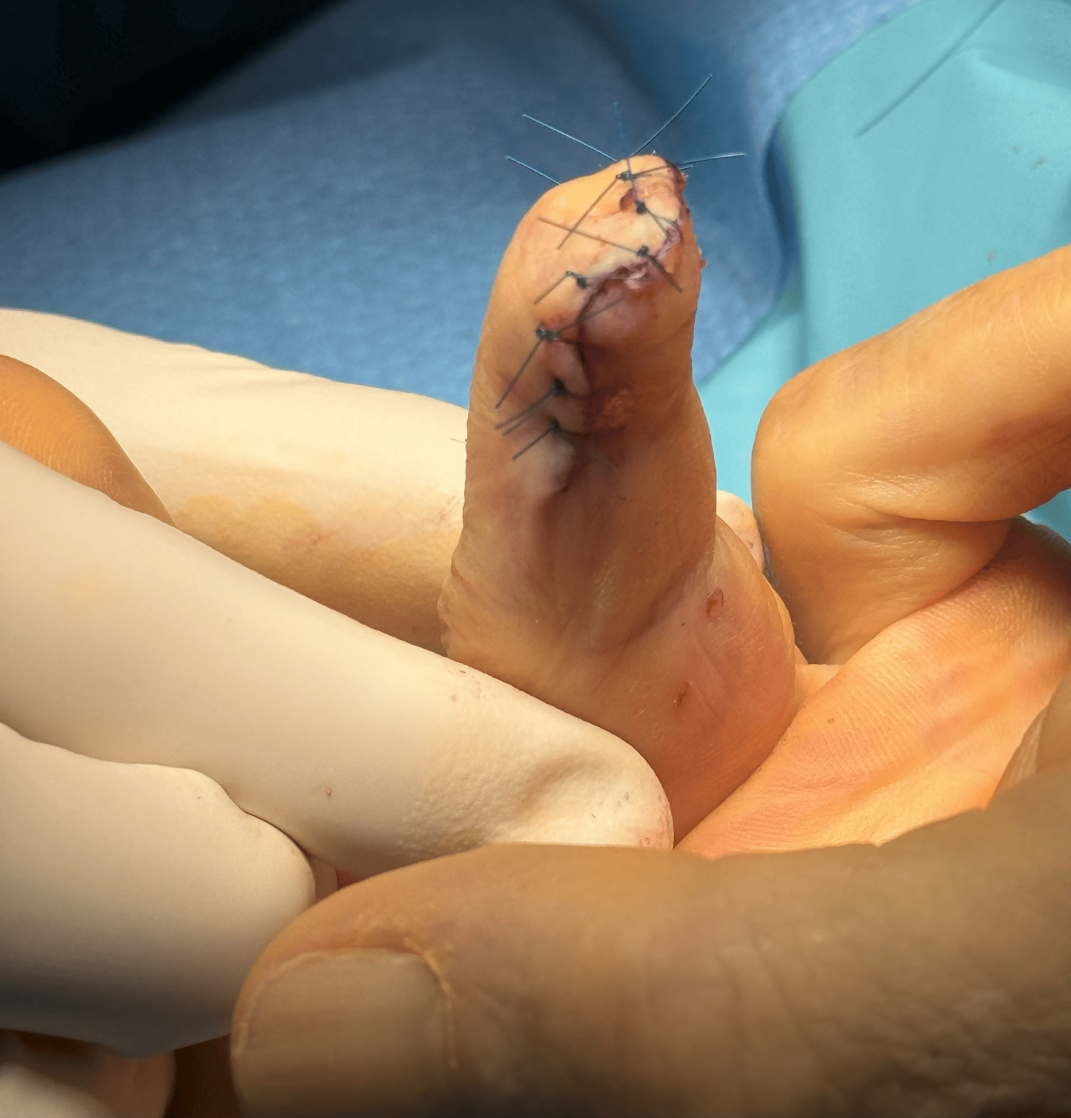
Surgical neurotomy and distal finger amputation in Case 1 patient.

Neither patient experienced any improvement in pain intensity, pattern, or functionality following treatment with BoNT-A. Numerical pain assessments remained unchanged during follow-up, and severe allodynia persisted eight months post-treatment. Both patients were subsequently referred for surgical neurotomy and distal finger amputation by the orthopedics department (Figure 4). Both patients achieved complete pain relief and remained pain-free three months post-surgery.

## Discussion

Neuropathic pain is a complex condition that remains challenging to treat due to its diverse pathophysiology and limited response to conventional therapies [7]. Pharmacological treatments often provide only partial relief, leaving many patients with unresolved pain [1]. Current management strategies typically combine pharmacological and non-pharmacological approaches tailored to individual patient needs.

First-line pharmacological options include gabapentinoids (gabapentin, pregabalin), which reduce neuronal excitability by modulating calcium channels [8], and serotonin-norepinephrine reuptake inhibitors (SNRIs) such as duloxetine and venlafaxine, which enhance descending inhibitory pain pathways [8]. Tricyclic antidepressants (TCAs) like amitriptyline and nortriptyline also show efficacy, likely due to their dual inhibition of serotonin and norepinephrine reuptake [8]. For localized neuropathic pain, topical agents such as lidocaine 5% patches and capsaicin 8% patches offer targeted relief by modulating peripheral nociceptor activity [9]. When first-line treatments are ineffective, second-line options include opioids like tramadol and tapentadol, which may benefit severe, refractory cases but carry risks of dependency and adverse effects [10]. Botulinum toxin type A (BoNT-A) has also been explored for select neuropathic syndromes, showing potential analgesic effects through inhibition of neurotransmitter release [11].

The rationale for investigating BoNT-A in trauma-induced neuropathic pain lies in its multifaceted mechanisms. BoNT-A inhibits the release of pain mediators (substance P, CGRP, and glutamate) and modulates sodium and calcium ion channels, potentially reducing peripheral sensitization and aberrant nerve firing. Its ability to undergo retrograde axonal transport suggests possible central effects on pain processing at the spinal level. BoNT-A has demonstrated efficacy in conditions like postherpetic neuralgia and diabetic neuropathy, where similar mechanisms are involved. We opted for BoNT-A due to the ease of its administration in injectable form directly into the painful area, in contrast to the challenges of applying capsaicin or lidocaine-impregnated patches on the fingertips.

Despite these promising mechanisms, this study found BoNT-A to be ineffective in two cases of trauma-induced neuropathic pain. The lack of response raises questions about its efficacy in certain neuropathic conditions. While BoNT-A has shown success in some cases, its primary mechanism - blocking acetylcholine release at the neuromuscular junction - may not effectively target the sensory neuron dysfunction underlying neuropathic pain. This highlights the need for careful patient selection and further research to better define BoNT-A’s role in neuropathic pain management.

A significant limitation of this study is its small sample size, which limits the generalizability of the findings. However, it offers valuable insight into the limitations of BoNT-A and supports further research across different neuropathic pain subtypes. The authors propose the "neuropathic finger model" as a standardized platform for evaluating injectable treatments for neuropathic pain. Due to the localized nerve structures and well-defined pain pathways of the fingers, this model allows for precise assessment of injectable therapies without the confounding effects of muscle involvement. Specifically for BoNT-A, the absence of muscle in the fingers permits a focused evaluation of its neural effects without the risk of muscle paralysis. In this study, BoNT-A was injected on both the medial and lateral sides of the affected fingers to comprehensively target sensory pathways involved in pain transmission.

Following the failure of BoNT-A, both patients underwent surgical neurotomy and phalangeal amputation, resulting in complete and sustained pain relief at the three-month follow-up. Neurotomy, which involves severing affected nerve pathways, is generally considered a last-resort option for severe, localized, and pharmacologically resistant neuropathic pain [12,13]. The successful outcomes in these cases suggest that neurotomy may have a role in managing focal neuropathic pain when conservative treatments fail. While it is uncertain if neuropathic pain could recur in these patients, the profound and debilitating nature of their pain justified the surgical approach. Although neurotomy carries risks such as residual pain, sensory deficits, neuroma formation, and phantom limb pain, its effectiveness in these cases warrants further investigation.

The positive outcomes observed in these cases highlight the importance of a multidisciplinary approach, where pharmacological, interventional, and surgical strategies are considered in sequence to optimize patient outcomes. Further studies are needed to refine patient selection criteria for BoNT-A and to evaluate the long-term efficacy and safety of surgical options like neurotomy in the management of refractory neuropathic pain.

## Conclusions

This study contributes to the limited evidence on BoNT-A for neuropathic pain (NP), concluding that it was ineffective in two cases of traumatic origin. Despite its proposed mechanisms of action, BoNT-A failed to provide pain relief, suggesting that its efficacy may be restricted to specific NP subtypes. The complete resolution of pain following surgical neurotomy reinforces its role as a viable option for refractory NP, particularly when conservative treatments fail. Furthermore, the authors suggest that the "neuropathic finger" may serve as a potential model for investigating local antineuropathic drugs.

## References

[1] Dekhne A, Goklani HD, Doshi N, Salian RB, Gandhi SK, Patel P (2023). Effectiveness of botulinum toxin in the treatment of neuropathic pain: a literature review. Cureus.

[2] Lagueny A, Burbaud P (1996). Mechanism of action, clinical indication and results of treatment of botulinum toxin. [Article in French]. Neurophysiol Clin.

[3] Lew MF (2002). Review of the FDA-approved uses of botulinum toxins, including data suggesting efficacy in pain reduction. Clin J Pain.

[4] Park J, Park HJ (2017). Botulinum toxin for the treatment of neuropathic pain. Toxins (Basel).

[5] Spagna A, Attal N (2023). Botulinum toxin A and neuropathic pain: an update. Toxicon.

[6] Schuler A, Veenstra J, Ozog D (2019). Battling neuropathic scar pain with botulinum toxin. J Drugs Dermatol.

[7] Attal N, Bouhassira D (2021). Advances in the treatment of neuropathic pain. Curr Opin Neurol.

[8] Finnerup NB, Attal N, Haroutounian S (2015). Pharmacotherapy for neuropathic pain in adults: a systematic review and meta-analysis. Lancet Neurol.

[9] Derry S, Wiffen PJ, Moore RA, Quinlan J (2014). Topical lidocaine for neuropathic pain in adults. Cochrane Database Syst Rev.

[10] Sommer C, Klose P, Welsch P, Petzke F, Häuser W (2020). Opioids for chronic non-cancer neuropathic pain. An updated systematic review and meta-analysis of efficacy, tolerability and safety in randomized placebo-controlled studies of at least 4 weeks duration. Eur J Pain.

[11] Attal N, de Andrade DC, Adam F (2016). Safety and efficacy of repeated injections of botulinum toxin A in peripheral neuropathic pain (BOTNEP): a randomised, double-blind, placebo-controlled trial. Lancet Neurol.

[12] Pessôa BL, Hauwanga WN, Thomas A, Valentim G, McBenedict B (2024). A comprehensive narrative review of neuropathic pain: from pathophysiology to surgical treatment. Cureus.

[13] Moreau N, Korai SA, Sepe G, Panetsos F, Papa M, Cirillo G (2024). Peripheral and central neurobiological effects of botulinum toxin A (BoNT/A) in neuropathic pain: a systematic review. Pain.

